# Targeted Long‐Read Sequencing as a Single Assay Improves the Diagnosis of Spastic‐Ataxia Disorders

**DOI:** 10.1002/acn3.70008

**Published:** 2025-02-25

**Authors:** Laura Ivete Rudaks, Igor Stevanovski, Dennis Yeow, Andre L. M. Reis, Sanjog R. Chintalaphani, Pak Leng Cheong, Hasindu Gamaarachchi, Lisa Worgan, Kate Ahmad, Michael Hayes, Andrew Hannaford, Samuel Kim, Victor S. C. Fung, Gabor M. Halmagyi, Andrew Martin, David Manser, Michel Tchan, Karl Ng, Marina L. Kennerson, Ira W. Deveson, Kishore Raj Kumar

**Affiliations:** ^1^ Molecular Medicine Laboratory and Neurology Department Concord Repatriation General Hospital Concord New South Wales Australia; ^2^ Faculty of Medicine and Health University of Sydney Camperdown New South Wales Australia; ^3^ Genomic and Inherited Disease Program The Garvan Institute of Medical Research Darlinghurst New South Wales Australia; ^4^ Clinical Genetics Unit Royal North Shore Hospital St Leonards New South Wales Australia; ^5^ Centre for Population Genomics The Garvan Institute of Medical Research Darlinghurst New South Wales Australia; ^6^ Neurodegenerative Service Prince of Wales Hospital Randwick New South Wales Australia; ^7^ Neuroscience Research Australia Randwick New South Wales Australia; ^8^ Faculty of Medicine University of New South Wales Kensington New South Wales Australia; ^9^ School of Computer Science and Engineering University of New South Wales Kensington New South Wales Australia; ^10^ Clinical Genetics Service Royal Prince Alfred Hospital Camperdown New South Wales Australia; ^11^ Neurology Department Royal North Shore Hospital St Leonards New South Wales Australia; ^12^ Movement Disorders Unit, Neurology Department Westmead Hospital Westmead New South Wales Australia; ^13^ Neurology Department Royal Prince Alfred Hospital Camperdown New South Wales Australia; ^14^ Department of Genetic Medicine Westmead Hospital Westmead New South Wales Australia; ^15^ The Northcott Neuroscience Laboratory ANZAC Research Institute, Sydney Local Health District Concord New South Wales Australia; ^16^ School of Clinical Medicine, Faculty of Medicine and Health, St Vincent's Healthcare Clinical Campus University of New South Wales Darlinghurst New South Wales Australia

**Keywords:** hereditary cerebellar ataxia, hereditary spastic paraplegia, nanopore sequencing, spinocerebellar ataxia

## Abstract

**Objective:**

The hereditary spastic‐ataxia spectrum disorders are a group of disabling neurological diseases. The traditional genetic testing pathway is complex, multistep and leaves many cases unsolved. We aim to streamline and improve this process using long‐read sequencing.

**Methods:**

We developed a targeted long‐read sequencing strategy with the capacity to characterise the genetic variation of all types and sizes within 469 disease‐associated genes, in a single assay. We applied this to a cohort of 34 individuals with unsolved spastic‐ataxia. An additional five individuals with a known genetic diagnosis were included as positive controls.

**Results:**

We identified causative pathogenic variants that would be sufficient for genetic diagnosis in 14/34 (41%) unsolved participants. The success rate was 5/11 (45%) in those who were naïve to genetic testing and 9/23 (39%) in those who were undiagnosed after prior genetic testing, completed on a clinical basis. Short tandem repeat expansions in *FGF14* were the most common (7/34, 21%). Two individuals (2/34, 6%) had biallelic pathogenic expansions in *RFC1* and one individual had a monoallelic pathogenic expansion in *ATXN8OS*/*ATXN8*. Causative pathogenic sequence variants other than short tandem repeat expansions were found in four individuals, including in *VCP*, *STUB1*, *ANO10* and *SPG7*. Furthermore, all five positive controls were identified.

**Interpretation:**

Our results demonstrate the utility of targeted long‐read sequencing in the genetic evaluation of patients with spastic‐ataxia spectrum disorders, highlighting both the capacity to increase overall diagnostic yield and to streamline the testing pathway by capturing all known genetic causes in a single assay.

## 1 Introduction

The hereditary spastic‐ataxia spectrum disorders are a group of rare, disabling neurologic conditions [1]. Hereditary cerebellar ataxia (HCA) and hereditary spastic paraplegia (HSP) can be considered as part of a spastic‐ataxia spectrum disorder [2]. Despite advances in genetic testing methods and broader access to next‐generation sequencing (NGS), up to 71% of individuals with HCA and 45%–50% of individuals with a HSP phenotype currently do not receive a genetic diagnosis [1, 3, 4, 5, 6, 7, 8].

There are several potential contributors to this ‘diagnostic gap’. For example, only the most common short tandem repeat (STR) expansions are evaluated as part of a ‘spinocerebellar ataxia (SCA) panel’, the content of which may vary between sites, while other less common or recently described STR expansions may be overlooked. For instance, presently in Australia, clinically accredited testing for STR expansions in *FGF14* or *RFC1*, which causes late‐onset ataxia [9], is not available locally.

Long‐read sequencing (LRS) is an emerging group of genomic technologies, which overcome several limitations posed by earlier methods, including NGS [10]. LRS provides improved detection of STR expansions, structural variants (SVs) and copy number variants (CNVs); the ability to resolve repetitive regions and homologous gene families or gene‐pseudogene pairs; variant phasing without parental sequencing data and DNA methylation profiling at no additional cost [10]. LRS is particularly advantageous for genotyping STR expansions, where it can clearly identify expansion size, sequence, methylation state and zygosity, even for large, complex or GC/AT‐rich repeats.

The adaptive sampling or ‘ReadUntil’ functionality on LRS instruments from Oxford Nanopore Technologies (ONT) enables selective sequencing of specific genes using genetic coordinates provided programmatically and without the need for additional laboratory processes for target enrichment [11]. Targeted LRS can be used to evaluate a large number of STRs in a single test and has demonstrated success in concurrent testing of a set of 37 genes associated with neurologic disease [12] as well as concurrent evaluation of 10 STR loci associated with ataxia [13]. Although these studies establish the analytical validity of this approach for detection of STR expansions, they did not examine non‐STR causative variants for ataxia/neurologic disease, nor assess the capacity of LRS to solve otherwise undiagnosed patients.

Given the capacity to evaluate a broad variety of genes and genetic variant types in a single streamlined assay, we reasoned that ONT‐targeted LRS could be used to improve the diagnosis of spastic‐ataxia spectrum disorders. To test this, we designed a targeted assay for the evaluation of STR expansions, single‐nucleotide variants (SNVs), small insertions and deletions (indels), SVs and CNVs across all diagnostically relevant genes (*n* = 469). We applied this to a cohort of genetically undiagnosed patients with a spastic‐ataxia spectrum phenotype and assessed the potential to improve the rate of diagnosis, relative to traditional testing. Here we report the clinical and genetic findings from this cohort, demonstrating a strong improvement in diagnostic rate and highlighting the strengths of LRS for genetic evaluation of these disorders.

## 2 Methods

### 2.1 Patient Recruitment, Clinical Evaluation and Study Approval

A cohort of 34 patients with clinically diagnosed spastic‐ataxia spectrum disorders, without a genetic diagnosis, were recruited from the Neuromuscular Clinic at Concord Repatriation General Hospital in Sydney, Australia, between April 2023 and March 2024. The cohort consisted of 11 individuals who were naïve to genetic testing and 23 individuals who had previously undertaken genetic testing, as per standard clinical practice in Australia. Prior genetic testing had been arranged by the patients' treating clinicians, and included various combinations of testing for STR expansions in selected SCA genes (SCA 1, 2, 3, 6, 7, 12, 17), *FXN* for Friedreich ataxia, *ATN1* for dentatorubral‐pallidoluysian atrophy, *FMR1* for Fragile X Tremor/Ataxia Syndrome (FXTAS); NGS [targeted gene panel, whole‐exome sequencing (WES) or whole‐genome sequencing (WGS)]; and auxiliary testing [single gene testing, multiplex ligation‐dependent probe amplification (MLPA), microarray, mitochondrial genome sequencing], as appropriate. An additional five individuals with spastic‐ataxia spectrum disorders and a known genetic diagnosis were included as positive controls. Demographic, clinical, neuroimaging, neurophysiological and genetic testing data were collected at the time of clinical evaluations and from patient healthcare records. The protocol for the study has received prior approval by the appropriate Institutional Review Board, and informed consent was obtained from each subject (approval number 2019/ETH12538).

### 2.2 Targeted Long‐Read Sequencing Workflow

Peripheral blood samples were obtained from all 39 individuals (34 study participants and five controls). Samples were processed at The Garvan Institute of Medical Research in Sydney, Australia. High‐molecular weight (HMW) genomic DNA was extracted from peripheral blood samples using the Nanobind CBB kit (PacBio, Cat# 102‐301‐900). HMW genomic DNA was sheared to ~30‐kb fragment size using the Diagenode Megaruptor 3 DNA shearing system and visualised on an Agilent Femto Pulse using the Genomic DNA 165 kb Kit. ONT libraries were prepared from ~3 μg of sheared HMW genomic DNA using a ligation prep (SQK‐NBD114.24). Three samples were barcoded and pooled into one library and loaded on an ONT PromethION R10.4.1 flow cell (FLO‐PRO114M) and sequenced on either a PromethION 2 Solo or PromethION 48 instrument, with live target selection/rejection executed by the Readfish software package (v0.0.10dev2) [11] targeting a custom panel of 469 genes associated with spastic‐ataxia spectrum disorders, plus the mitochondrial genome (Table S1). Gene targets were encoded relative the T2T‐chm13 (v2) reference genome and ReadFish was executed with the following configurations: config_name = ‘dna_r10.4.1_e8.2_400bps_5khz_fast_prom’; min_chunks = 0; max_chunks = 16; single_on = ‘stop_receiving’; multi_on = ‘stop_receiving’; single_off = ‘unblock’; multi_off = ‘unblock’; no_seq = ‘proceed’; no_map = ‘proceed’. Genes included in the targeted panel were selected via detailed literature review [9] and review of gene panels for ataxia, spastic‐ataxia and HSP through PanelApp [14] and other genetic testing laboratories including Invitae, Blueprint Genetics, PathWest and Victorian Clinical Genetics Services (VCGS). Libraries were run for 72 h, with nuclease flushes and library reloading performed at approximately 24‐ and 48‐h time points.

### 2.3 Analysis of Genetic Variation

After completing a targeted sequencing run, raw ONT sequencing data were converted to BLOW5 format [15] and base‐called with Dorado, using the ‘super‐accuracy’ model (dna_r10.4.1_e8.2_400bps_5khz_sup_prom.cfg) and the Buttery‐eel wrapper to enable BLOW5 input [16]. The resulting ‘pass’ FASTQ files were aligned to both the hg38 and T2T‐CHM13v2.0 reference genomes using minimap2 (v2.14‐r883) [17]. SNVs and indels were called using Clair3 (v1.0.4) [18] and phased using WhatsHap (v2.1) [19]. SVs (including CNVs) were called with Sniffles2 [20] (v2.07), which takes haplotagged BAM alignment files to generate phased SV calls. SNVs and indels were functionally annotated using variant effect predictor (VEP) [21] (v110) and SVs were annotated using AnnotSV [22] (v3.3.4).

Variants were prioritised for further consideration based on the following criteria: (i) those annotated in ClinVar with a clinical significance of ‘pathogenic’, ‘likely pathogenic’ or ‘uncertain’; (ii) variants predicted to cause a frameshift; (iii) missense variants with a REVEL score greater than 0.5 and classified as ‘pathogenic’, ‘likely pathogenic’, ‘uncertain’ or without an assigned clinical significance and (iv) variants with high or moderate impact that are similarly classified as ‘pathogenic’, ‘likely pathogenic’, ‘uncertain’ or lacking an assigned clinical significance in ClinVar. All pre‐selected variants were within MANE Select transcripts and either were not found in gnomADv4 or had allele frequency of less than 0.1, resulting in a list of rare and potentially causative variants. Additionally, SVs intersecting protein‐coding genes were prioritised for further consideration.

### 2.4 Analysis of STR Expansions

We analysed STRs in 21 genes associated with spastic‐ataxia spectrum disorders (Table S2). During this project, STR expansions in *THAP11* and *ZFHX3*, were identified to cause HCA [23, 24, 25], and these genes were subsequently added to our target panel. All individuals underwent sequencing of *THAP11*, however, 24 samples had already undergone sequencing prior to the addition of *ZFHX3*, and therefore, these individuals were not evaluated for SCA4. Furthermore, *TBP* was omitted from the initial panel, and therefore also was only evaluated in 15/39 samples.

**FIGURE 1:**
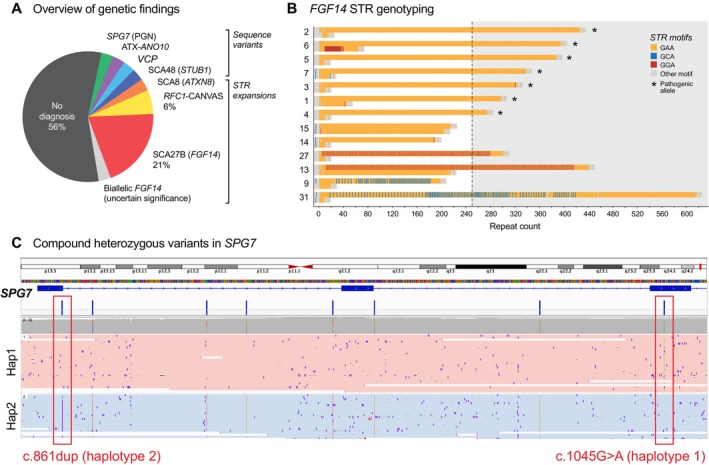
Improved diagnosis of spastic‐ataxia spectrum disorders with targeted long‐read sequencing. (A) Summary of genetic diagnoses in our cohort of 34 genetically undiagnosed patients with spastic‐ataxia spectrum disorders. (B) Sequence bar charts show STR alleles identified in 13 patients with expanded STRs in *FGF14*. Seven individuals, denoted with asterisks, had pure (GAA) expansions longer than 250 copies, sufficient for SCA27B diagnosis. The remaining six patients had (GAA) expansions that did not reach 250 copies or expanded (GAA) repeats interrupted by other motifs (GCA or GGA), not considered pathogenic. (C) Genome browser view shows detection of two pathogenic heterozygous variants within *SPG7* in a single patient. Alignments are phased into separate haplotypes (pink = haplotype 1; blue = haplotype 2), confirming the two pathogenic variants are on alternative haplotypes (i.e., *in trans*).

STRs were genotyped using a method demonstrated previously [12]. Each STR region was first inspected in integrated genome viewer (IGV; T2T‐CHM13v2 reference) [26]. For sites showing evidence of expansions (i.e., large insertions within alignments spanning an STR site), reads were retrieved within a 50 kb window centred on the target STR and assembled *de novo* with Flye (v2.8.1‐b1676) [27] to create a pseudo‐haploid contig encompassing the STR region. The starting reads were then realigned to this contig and phased into separate haplotypes using Longshot (v0.4.1) [28]. The initial assembled contig was re‐polished with reads from each haplotype using Racon (v1.4.0), generating two distinct haploid contigs encompassing the STR site. The precise position of the STR site was identified by mapping 150‐bp unique flanking sequences extracted from T2T‐CHM13v2.0 using minimap2 (v2.22). The reads from each haplotype were mapped to their respective polished contigs and re‐inspected in IGV to identify potential discrepancies in phasing of reads, which could be manually corrected before re‐polishing [26]. The intervening distance between unique mapped flanking sequences was used to determine the size of the STR on each haplotype, and sequences were extracted to determine the STR motif present, and/or presence of interruptions (where relevant). Custom sequence bar plots were created in Prism to enable visualisation of STR alleles within and between patients, with multi‐read bar plots used to verify the presence of interruptions or noncanonical motifs (see Figure 1 and Figure S2B for examples).

### 2.5 Validation of Results

Our analysis method for genotyping STR expansions with ONT data has previously been validated in a cohort of 37 individuals including patients with neurogenetic diseases, premutation carriers and controls [12]. In the present study, five individuals with a known genetic diagnosis were included as positive controls: one individual with FXTAS due to an *FMR1* STR expansion; one with SCA3 due to an *ATXN3* STR expansion, one with HSP due to homozygous *SPG7* variants; one with recessive ataxia due to compound heterozygous variants in *ANO10* and one with recessive ataxia due to compound heterozygous variants in *TDP2*. Novel findings were confirmed on orthogonal testing in nine individuals.

### 2.6 Statistical Analysis

Quantitative variables were described as means and ranges. Categorical variables were expressed with frequencies and percentages. Comparison of groups was undertaken using independent samples 𝜒^2^ testing for binomial variables. Statistical significance threshold was set at *p*‐value < 0.05.

### 2.7 Data Avalability Statement

Anonymised data that support the findings of this study are available from the corresponding author, upon reasonable request.

## 3 Results

### 3.1 Targeted LRS Assay for Spastic‐Ataxia Spectrum Disorders

We developed an ONT ReadUntil targeted LRS assay [11] designed to capture the full suite of genetic features implicated in spastic‐ataxia spectrum disorders in a single assay. This encompasses 469 genes considered diagnostically relevant plus the mitochondrial genome (Table S1). Genes included in the targeted panel were selected via detailed literature review [9], PanelApp [14] and review of gene panels from other genetic laboratories. We designed target regions covering each gene and flanking regions 50 kb upstream and downstream to ensure all exons, introns, gene promoters, untranslated regions (UTRs) and other local regulatory sequences are captured. This panel covers STR sites in 21 genes where expanded alleles are known to cause spastic‐ataxia spectrum disorders (Table S2). In total, the target panel covers 91.8 Mbases or 3.0% of the human genome sequence.

Targeted LRS enables multiple patient samples to be analysed in a single experiment to reduce cost, while still obtaining sufficient coverage depth for accurate and comprehensive analysis. Our study cohort (see below) was sequenced at a rate of three patients per flow cell (ONT PromethION), which equates to an approximately threefold reduction in cost per patient, relative to whole‐genome ONT sequencing. We obtained median 42‐fold coverage depth across target gene regions, with a median absolute deviation (MAD) of 16.79. The coverage depth across individual target regions ranged from a minimum median coverage of 23 to a maximum median coverage of 50 (Figure S1A,B). For on‐target statistics per sample, see Table S3, for average mitochondrial DNA coverage and read length see Table S4.

To validate our approach, positive controls were analysed by blinded investigators (see Materials & Methods). Our targeted ONT LRS approach identified STR expansions in *FMR1* and *ATXN3* in patients with known FXTAS and SCA3, respectively, with relatively similar repeat lengths identified compared to that generated by standard clinical testing (85 vs. 87 copies for *FMR1*; 73 vs. 76 for *ATXN3*; Figure S2A,B). Additionally, ONT LRS was able to identify the presence and position of two AGG interruptions within the *FMR1* CGG STR expansion (Figure S2B). AAG interruptions are known to influence the propensity of the *FMR1* CGG STR expansion to further expand in subsequent generations [29]. We identified SNVs and small indels in three positive control patients with recessive spastic ataxic disorders (Figure S2A,C). Our ONT LRS approach also allowed for phasing of these variants, confirming an apparently homozygous SNV in *SPG7* was biallelic and confirming that patients with two heterozygous variants in *ANO10* and *TDP2* carried these variants *in trans* (Figure S2C). Interestingly, the patient with known autosomal recessive SCA due to variants in *TDP2* was also found to carry a (GAA)_240_ STR expansion in *FGF14*, close to the (GAA)_250_ threshold considered pathogenic.

### 3.2 Cohort Characteristics

We evaluated the performance of our targeted LRS strategy on a cohort of 34 patients (Table 1) with genetically undiagnosed spastic‐ataxia spectrum disorders, in addition to five positive controls (see above).

**TABLE 1:** Baseline characteristics of patients with genetically undiagnosed spastic‐ataxia spectrum disorders.

Characteristic	Mean (years) (range)
Age at examination	64.0 (24–84)
Age at onset	53.0 (0.08–80)
Disease duration	10.9 (1–37)
Sex	***n* (%)**
Female	16 (47)
Male	18 (53)
Ancestry	
White/European	20 (59)
Lebanese	2 (6)
Chinese	1 (3)
Filipino	1 (3)
Indian	1 (3)
Iraqi	1 (3)
Mixed	2 (6)
Unknown	6 (18)
Family history	
Sporadic	20 (59)
AD	11 (32)
AR	1 (3)
Unknown	2 (6)
Predominant phenotype	
Ataxia	29 (85)
Spastic‐ataxia	3 (9)
Spastic paraparesis	2 (6)

Abbreviations: AD, autosomal dominant; AR, autosomal recessive.

### 3.3 Genetic Findings

Our targeted LRS strategy identified a causative pathogenic variant sufficient for diagnosis in 14/34 (41%) of participants (Figure 1A). In the group who had previously undergone unsuccessful genetic testing, a diagnosis was obtained in 9/23 (39%) participants, while a diagnosis was obtained in 5/11 (45%) who were naïve to genetic testing. A heterozygous GAA‐*FGF14* STR expansion ≥ 250 repeats, consistent with a diagnosis of SCA27B, was identified in 7/34 (21%) of the study cohort and was found in 5/23 (22%) of the testing‐negative group (Figure 1B). *RFC1* CANVAS/spectrum disorder due to biallelic *RFC1* STR expansions was identified in 2/34 (6%). A single individual was identified to have each of: a CAG·CTG STR expansion in *ATXN8/ATXN8OS*; a homozygous splicing variant in *ANO10* (c.1163‐9A>G); a heterozygous missense variant in *VCP* (c.475C>T, p.Arg159Cys); a heterozygous in‐frame deletion in *STUB1* (c.433_435del, p.Lys145del); one instance of compound heterozygous missense and nonsense variants in *SPG7* (c.1045G>A, p.Gly349Ser and c.861dup, p.Asn288Ter; Figure S3A–D). For the latter case, variants were phased with LRS without parental sequencing data. The 75.7 kb phased block contained six additional heterozygous variants between the two variants of interest, supported by 23 long reads, providing robust evidence to establish compound heterozygosity (Figure 1C). Genetic and clinical features for individuals with a positive diagnosis are summarised in Table S5.

This left 20/34 (59%) of individuals without a genetic diagnosis. Two of these individuals were found to harbour pathogenic/likely pathogenic variants in *GCH1* and *PRRT2*, respectively, although these findings were of uncertain clinical relevance to the cerebellar ataxia phenotype (File S1). A further individual was identified to have biallelic STR expansions in *FGF14*, within the range of 200–249 repeats (File S1), of uncertain clinical relevance. Overall, one or more variants of uncertain significance (VUS) were identified in 6/20 (30%) of individuals without an identified causative variant (Table S6).

To validate our findings, eight individuals (patients 1, 3, 4, 5, 7, 8, 12 and 13) underwent confirmatory testing through an independent clinically accredited laboratory. One further individual (patient 11) had previously undergone WGS on a research basis and data were available for reanalysis. Five individuals with *FGF14* STR expansions, as well as one individual with biallelic *RFC1* STR expansions, underwent confirmatory testing with standard flanking PCR and repeat‐primed PCR, followed by capillary electrophoresis. Results demonstrated similar GAA repeat lengths between the two methods and in all cases, the pathogenicity of expansions was correctly classified (Table S7). The biallelic *RFC1* AAGGG STR expansion identified in patient 8 was also confirmed with standard flanking PCR and repeat‐primed PCR followed by capillary electrophoresis, although the specific repeat length was not reported. The homozygous *ANO10* variant identified in patient 12, and the heterozygous *VCP* variant identified in patient 13 were confirmed on clinically accredited WES. The *STUB1* in‐frame deletion in patient 11 was confirmed on the retrospective review of prior research WGS data, and further confirmatory testing with a clinically accredited WES is pending.

### 3.4 Clinical Findings

#### 3.4.1 *FGF14* ‐GAA (SCA27B); demographic and Clinical Features

**FIGURE 2:**
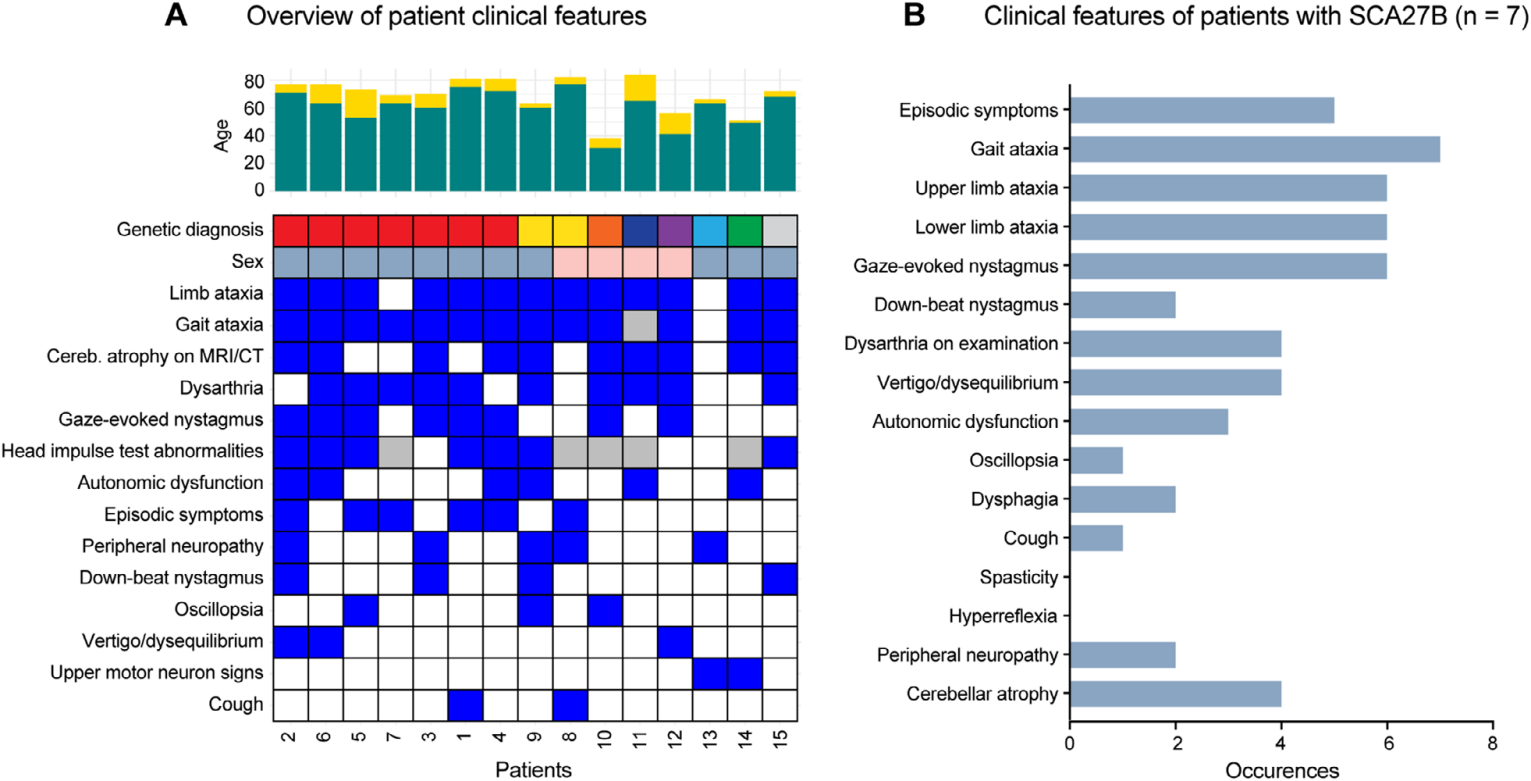
Clinical features of spastic‐ataxia patients receiving a genetic diagnosis via long‐read sequencing. (A) Chart provides an overview of clinical characteristics for all patients who received a genetic diagnosis (plus patient 15, with biallelic *FGF14* GAA expansions < 250 repeats, of uncertain clinical significance). The upper bar chart shows patient ages at time of analysis (yellow) and their age at symptom onset (green). The heatmap below indicates symptoms identified in each patient (blue = positive; white = negative; grey = not assessed). Patient sex is encoded with blue (male) or pink (female) tiles and genetic diagnoses are coded as follows: *FGF14* = red; *RFC1* = yellow; *ATXN8* = orange; *STUB1* = navy; *ANO10* = purple; *VCP* = blue; *SPG7* = green; biallelic *FGF14* (uncertain clinical significance) = grey. Patients with SCA27B are sorted by *FGF14* expansion size, in matched order to Figure 1B. (B) Bar chart summarises the frequency of clinical phenotypes observed among patients diagnosed with SCA27B, based on STR expansions in *FGF14* (*n* = 7 patients).

A total of 7/34 (21%) individuals were identified to have GAA ≥ 250 *FGF14* STR expansions, consistent with a diagnosis of SCA27B. The mean GAA repeat length was 348 repeats (range 274–425, SD ±56) (Figure 1B, Videos S1 and S2). All seven individuals were male. Ancestry was known in 6/7 (86%) individuals, with white/European ancestry in all known cases. An autosomal dominant family history was apparent in 3/7 (43%) of individuals, with maternal inheritance in all cases. The mean age at examination was 75.4 years (range 69–81), the mean age of onset was 65.3 years (range 53–75) and the mean disease duration was 10.1 years (range 6–20). 4/7 (57%) required a mobility aid (stick 2/4 and walker 2/4), with a mean time to mobility aid requirement of 5.8 years from disease onset (range 4–10). The mean SARA score was 11.6 (range 6.5–18). Clinical features of all genetically diagnosed patients, and those with SCA27B specifically, are indicated in Figure 2A,B.

#### 3.4.2 Non‐pathogenic *FGF14* STR Expansions

Four individuals were identified to have noncanonical interrupted GAA *FGF14* STR expansions, three of which were ≥ 250 copies in length (Figure 1B). The longest was a mixed GAA/GCA expansion of 616 repeats, with a maximal uninterrupted GAA length of 195 repeats, while two patients had mixed GAA/GGA repeats with total repeat lengths of 440 and 330 respectively. These findings were not considered pathogenic as none of these noncanonical alleles contained an uninterrupted stretch of ≥ 250 GAA copies.

Two further participants, as well as one of the positive controls, were found to have GAA repeats in the 200–249 range (Figure 1B). Patient 13 had a STR expansion of 213 pure GAA repeats, in addition to the nonpathogenic mixed 440 GAA/GGA repeat. This individual was identified to have a pathogenic variant in *VCP*, with a matching phenotype, and the *FGF14* GAA_213_ STR expansion was not felt to be contributory to his presentation. The other individual was identified to have a biallelic *FGF14* GAA repeat expansion, with 213/201 repeats, which may contribute to their phenotype. This case is discussed in File S1.

#### 3.4.3 *RFC1* ‐CANVAS; Clinical and Genetic Features

*RFC1*‐CANVAS was identified in two individuals. Both presented with the full CANVAS syndrome including cerebellar ataxia, neuropathy and vestibular areflexia, with additional clinical features indicated in Table S5. Patient 8 was of white/European background and had biallelic (AAGGG)_n_ expansions, with 651 and 693 repeats. The second individual (patient 9), from the Philippines, was compound heterozygous for an (AAGGG)_1000_ expansion and an (ACAGG)_2000_ expansion.

### 3.5 Clinically Relevant Sequencing Variants

We identified clinically relevant sequencing variants in the *VCP* and *STUB1* genes in unsolved participants (Figures 1A and 2A).

The *VCP* variant carrier (patient 13), a 66‐year‐old man of white/European ancestry, presented with features of complex HSP (Table S5). He had a history of osteoporosis, migraines and had previously suffered a right cerebellar stroke, with complete symptomatic recovery. There were no cognitive symptoms. Family history revealed that his father had mobility issues and dementia, and his brother had frontotemporal dementia. MRI demonstrated a small right chronic cerebellar infarct. A dual‐phase whole‐body bone scan did not demonstrate scintigraphic evidence of Paget's disease. ONT LRS identified a pathogenic missense variant in *VCP* (c.475C>T, p.Arg159Cys). This was confirmed on clinically accredited WES and has been previously reported [30].

Patient 11, the *STUB1* variant carrier, an 84‐year‐old lady of German/European ancestry presented with autosomal dominant ataxia, with similar features reported in her mother and mild features in her daughter. Clinical features are indicated in Table S5. ONT LRS identified an in‐frame deletion in *STUB1* (c.433_435del). Prior SCA17 testing had identified normal *TBP* repeat length (35/36 repeats). The patient had previously undergone WGS, and data were retrospectively reviewed, with confirmation of the *STUB1* variant. Since the time WGS was originally undertaken, additional cases with the same *STUB1* variant have been published, permitting classification of the variant as likely pathogenic [31, 32].

## 4 Discussion

**FIGURE 3:**
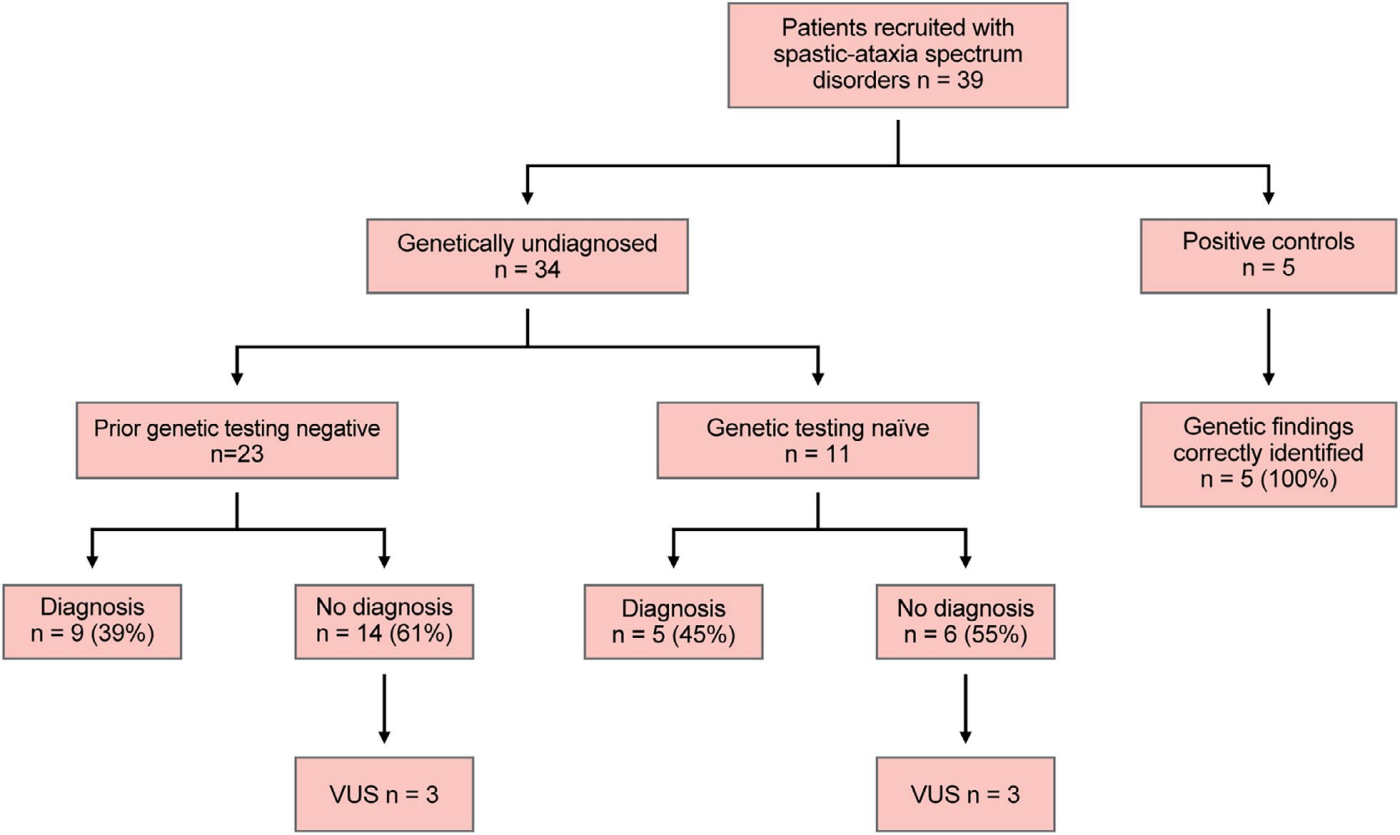
Overview of outcomes. Flow chart provides a schematic overview of diagnostic outcomes across the study cohort, which included 34 genetically undiagnosed patients with spastic‐ataxia spectrum disorders and five patients with known genetic diagnoses included as positive controls.

Our study evaluated 34 patients with genetically undiagnosed spastic‐ataxia spectrum disorders using targeted ONT LRS and identified a likely diagnosis in 14/34 (41%; summarised in Figure 3). In the group who had not previously undergone genetic testing, the diagnostic yield was 45% (5/11). In those who had previously had a genetic evaluation, with no diagnosis found, ONT LRS identified a cause in 39% (9/23). The high success rate in the latter group demonstrates the potential for LRS to greatly improve the rate of genetic diagnosis in these disorders.

There are multiple explanations for the diagnostic uplift achieved by our LRS assay. STR expansions account for most diagnoses in the cohort (10/14, 71%). The most common diagnosis was SCA27B, identified in 7/34 (21%), while two individuals (6%) were identified to have *RFC1*‐CANVAS. These two STR expansions were both only relatively recently identified [33, 34, 35], and currently, testing in Australia is only offered on a research basis. Lack of access to clinical testing combined with the relatively high frequency of *FGF14* and *RFC1* STR expansions in patients with HCA underpins the high rate of detection of these two variants within our cohort. Another individual was diagnosed with SCA8, caused by an STR expansion in *ATXN8*/*ATXN8OS* [36]. While clinical testing is available, this is one of several rare STR expansions that are not universally incorporated into standard genetic testing pathways, or routine ‘SCA panels’ so may be overlooked, as was the case in our reported patient. The ability to identify these unexpected diagnoses is an advantage of our integrated approach that captures all relevant STRs on every test. The remaining 4/14 (29%) of diagnoses were accounted for by conventional sequence variants (SNVs and indels). Two of these patients had undergone previous genetic testing, without identification of the causative variant. One patient was diagnosed with SCA48 caused by a variant in *STUB1*, which was not recognised as pathogenic at the time of previous genetic evaluation due to the lack of recognition of dominant inheritance at the time of initial analysis [37]. The other was found to have a variant in *VCP*; this gene was not included for evaluation on a prior HSP panel. A further patient, who was genetic testing naïve was identified to have a compound heterozygous variant in *SPG7*, which could not be phased using short‐read NGS to confirm pathogenicity. The capacity of LRS to phase autosomal recessive or *de novo* variants or identify both an STR expansion and sequence variant in a compound heterozygous state (as can occur in *RFC1‐*CANVAS/spectrum disorder and Friedreich ataxia [38, 39]), is especially valuable for late‐onset conditions, where availability of both parents for testing may be a barrier to diagnosis.

Pathogenicity of STR expansions may depend not only on repeat length, but on the repeat motif composition and the presence of interruptions. For example, *RFC1‐*CANVAS/spectrum disorders are usually caused by biallelic (AAGGG)_n_ STR expansions, but there are other pathogenic motifs, including (ACAGG)_n_, as well as several nonpathogenic motifs, including (AAAAG)_n_, (AAGAG)_n_, (AAAGGG)_n_ and smaller (AAAGG)_n_ expansions [31]. In such disorders, it is vital to obtain both repeat length and sequencing data, to confirm a diagnosis. Clinical testing may be undertaken with a combination of flanking PCR and repeat‐primed PCR, which will identify specific common repeat motif(s), but will typically not detect the less common motifs, requiring further testing for such cases [40, 41]. In contrast, LRS can detect and characterise the various repeat motif types, as well as repeat length in a single test. For example, patient 9, with *RFC1‐*CANVAS, was identified to have both an (AAGGG)_n_ and an (ACAGG)_n_ expansion. Furthermore, several individuals were found to have non‐pure GAA *FGF14* STR expansions. In these cases, LRS provided the capacity to quantify the maximum pure GAA repeat length, which fell below the 250‐repeat threshold for pathogenicity in all cases [42]. This highlights the strong utility of LRS for the evaluation of STR expansions, being the only technique that can identify size, motif composition, zygosity, presence of interruptions, flanking variation and methylation status in a single assay [12].

The ability to capture the full variety of genomic features potentially implicated in spastic‐ataxia spectrum disorders in a single assay not only improves diagnostic yields but can greatly streamline the diagnostic pathway. In clinical practice, STR expansions are commonly evaluated with repeat‐primed PCR and/or Southern blot, which require separate assays and/or specific primers/probes for each different gene [12]. In addition to requiring multiple sequential tests for some patients, this also incurs delays in the availability of clinical testing for newly described STR expansions as each new gene‐specific assay must be developed and validated [5]. In contrast, new genes and/or STR targets can be added to an ONT adaptive sampling assay simply by updating the set of genome targets provided programmatically during sequencing, requiring no change to either the upstream laboratory processes or downstream bioinformatics analysis pipelines [18]. Therefore, ONT LRS facilitates adaptability and rapid addition of new genes as they are discovered. This was exemplified in our study, as during the project, two additional ataxia‐associated STR expansions were identified; *THAP11* (‘SCA51’) [23, 43] and *ZFHX3* (recently identified as the basis of SCA4) [24, 25]. Both genes were subsequently added to our gene panel. However, 24 samples had already undergone sequencing and were not evaluated for STR expansions in *ZFHX3*. Conversely, this also brings to light a limitation of targeted ONT LRS, which is the inability to reanalyse data for variants at additional ‘off‐target’ loci, if new genes are discovered. This would be negated using whole‐genome LRS, with either ONT or Pacific Biosciences (PacBio) technology but would come at an additional cost per patient.

Despite the described benefits of LRS, there remain several limitations and obstacles to the clinical uptake of this technology. On a read‐by‐read level, accuracy for detecting SNVs and small insertions/deletions (indels) is lower than NGS [44]. Targeted ONT LRS can identify 98.8% of SNVs, although greater inaccuracy is seen in samples with reduced depth of coverage [44]. Detection of indels suffers from a greater level of inaccuracy, on account of an increased propensity for slippage errors [11]. Software tools are less well‐developed for data analysis, mapping and variant calling compared to NGS [10]. Due to the need for HMW DNA, freshly collected samples and specialised DNA extraction procedures are generally required [10]. In our study, fresh blood samples were generally provided for HMW DNA extraction and sequencing within 48 hours of collection, ensuring high‐quality DNA could be obtained. Where samples are not freshly collected and/or properly stored (−80°C), or where HMW DNA extraction protocols are not used, the presence of shorter DNA fragments is likely to adversely impact both the sequencing yield and read‐lengths obtained, both of which can impact detection of difficult variants like SVs, STR expansions, etc. Other important variables impacting sequencing yields per patient in our study were the initial ONT flow cell quality (MUX scan) and the ability to achieve precise balance in the molarity of different patient samples multiplexed on a single flow cell, which determines the relative sequencing yield on each sample. Finally, the cost of LRS remains higher than short‐read NGS, although pricing has, and is predicted to continue to improve [10].

Beyond technology‐related factors, other limitations of this study include potential selection bias. Patients were recruited from a tertiary centre, receiving referrals predominantly from primary care physicians and neurologists, with recruitment undertaken by authors L.I.R, D.Y. and K.R.K., neurologists, with subspecialty experience in neurogenetic disorders. The application of study protocols to broader neurology clinics could potentially impact the cohort characteristics and diagnostic rate. Most individuals in our cohort were of white/European background, and therefore results may differ when applied to broader population groups. Furthermore, although patients with features consistent with any spastic‐ataxia spectrum disorder were eligible for the study, 29/34 (85%) had a primary HCA phenotype, while only two individuals had a primary HSP phenotype. This was likely contributed to by referral patterns, with several HCA patients referred to our research centre by local neurologists aware of this study, as well as the commencement of recruitment in late 2023 for a concurrent study, for which individuals with a primary HSP phenotype were eligible.

After undergoing genetic evaluation, many individuals with spastic‐ataxia spectrum disorders remain without a genetic diagnosis, which has implications for clinical care. The provision of a genetic diagnosis may terminate a prolonged and arduous diagnostic odyssey, avoid further unnecessary and invasive investigations, permit disease‐specific management and provide more accurate prognostic information. Although only few gene‐specific treatments are presently available (e.g., treatment with 4‐aminopyridine for SCA27B) [45], eligibility for clinical trials may also be affected by a genetic diagnosis. Furthermore, there are important implications for reproductive choices and family counselling, with the ability to offer genetic testing in at‐risk family members [46]. Evidently, there is clear impetus to improve current genetic testing methods, to close this diagnostic gap. Our study begins to address this gap through the development and evaluation of a targeted LRS strategy that accurately identifies all classes of genomic variation across a panel of genes implicated in spastic‐ataxia spectrum disorders. A sizeable fraction of the diagnoses we made were not STR expansions and would have been missed using previously published LRS approaches for ataxia [13]. Therefore, our approach represents a clinically meaningful advance by being more comprehensive compared to LRS methods focused primarily on the detection of repeat expansion disorders. This integrated ‘single test’ approach is now poised to replace fragmented genetic testing regimes, leading to improved diagnostic rates and streamlining the diagnostic pathway for patients.

## Author Contributions

L.I.R., I.W.D., and K.R.K. contributed to conception and design of the study. L.I.R., I.S., D.Y., A.L.M.R., S.R.C., P.L.C., H.G., L.W., K.A., M.H., A.H., S.K., V.S.C.F., M.H., A.M., D.M., M.T., K.N., M.L.K., I.W.D., and K.R.K. were involved in acquisition and analysis of data. L.I.R., I.S., D.Y., A.L.M.R., S.R.C., I.W.D., and K.R.K. drafted a significant portion of the manuscript or figures.

## Conflicts of Interest

The authors declare no conflicts of interest.

## Supporting information


**Figure S1.** Sequencing coverage metrics for targeted long‐read sequencing assay.

**Figure S2.** Accuracy of targeted long‐read sequencing confirmed by analysis of positive control cases.

**Figure S3.** Detection and phasing of pathogenic sequence variants in four undiagnosed participants.

**Table S1.** Custom panel of genes evaluated in patients with spastic‐ataxia spectrum disorders.

**Table S2.** Genes associated with spastic‐ataxia spectrum disorders evaluated for short tandem repeat expansions.

**Table S3.** Average read length and coverage depth for spastic‐ataxia genes in individual participant samples.

**Table S4.** Average read length and coverage depth for mitochondrial DNA in individual participant samples.

**Table S5.** Clinical features and genetic findings for patients with a causative variant.

**Table S6.** Variants of uncertain significance in participants without an identified causative/pathogenic variant.

**Table S7.** *FGF14* STR expansion lengths on LRS and confirmatory flanking/repeat‐primed PCR.

**Video S1.** Cranial nerve examination in individual 3 with an *FGF14* GAA expansion (321/9 repeats) showing gaze evoked and downbeat nystagmus and cerebellar dysarthria. Upper limb examination reveals bilateral past pointing and impaired finger chase. Gait examination shows a broad‐based ataxic gait requiring a four‐wheel rollator walking frame.

**Video S2.** Cranial nerve examination in individual 5 with an *FGF14* GAA expansion (387/9 repeats) shows gaze‐evoked nystagmus. Upper limb examination reveals bilateral past pointing. Lower limb examination shows mild heel shin ataxia (more apparent when examined lying supine). Gait examination shows a very unsteady gait requiring one‐person support. He wears a helmet due to frequent falls and reports an improvement in ataxia with sleep.

**File S1.** Genetic and clinical features for participants with nondiagnostic findings and variants of uncertain significance.

## Data Availability

Data is available on request.
