# Supplementary material for: Targeted Long‐Read Sequencing as a Single Assay Improves the Diagnosis of Spastic‐Ataxia Disorders
Source: Ann Clin Transl Neurol. 2025 Feb 25;12(4):832–41. doi: 10.1002/acn3.70008 (PMC12040508; doi:10.1002/acn3.70008)
Supplement: Supplementary file 1 — Figure S1. Sequencing coverage metrics for targeted long‐read sequencing assay. [file ACN3-12-832-s001.pdf]

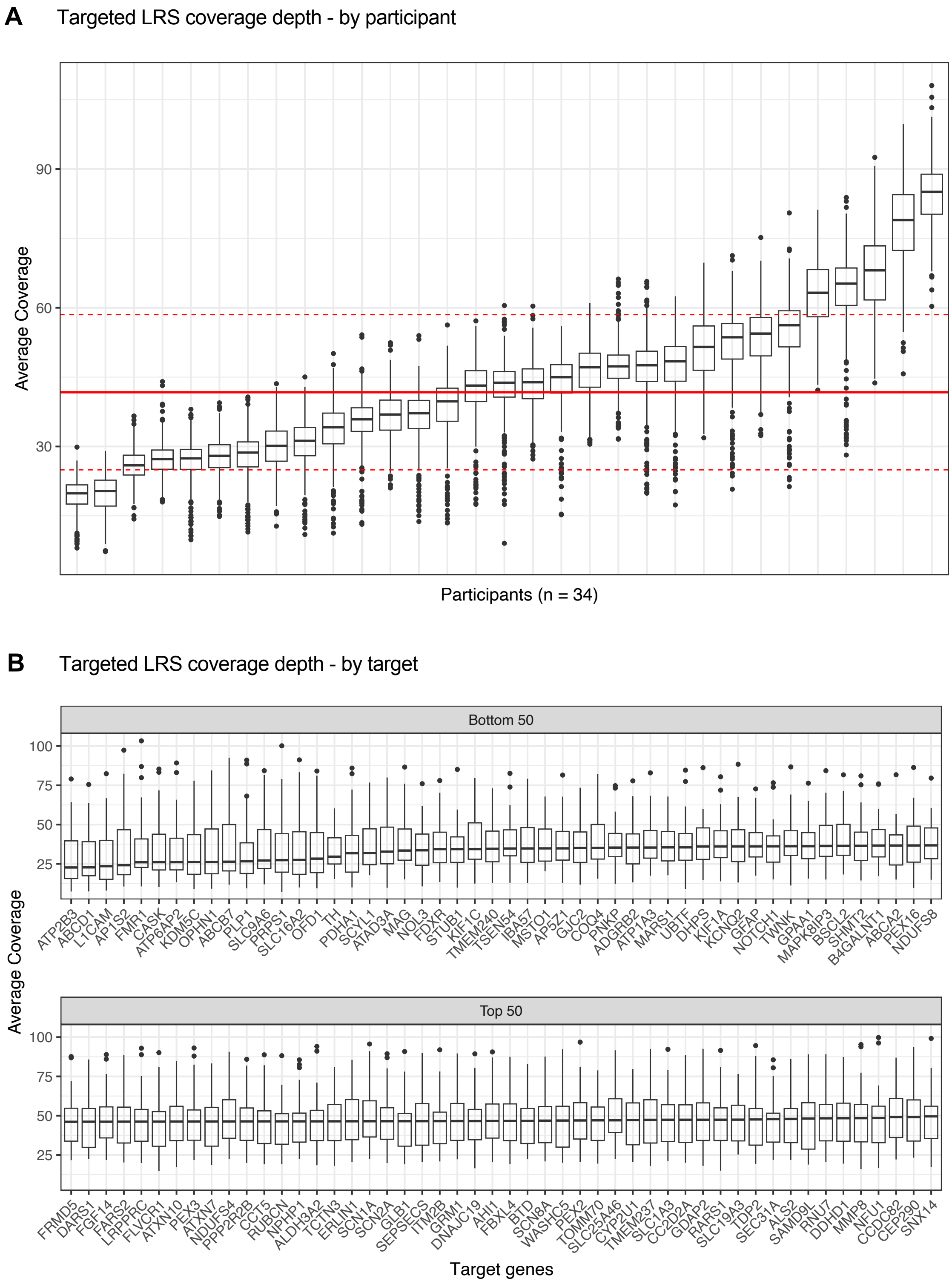

Supplementary Figure 1. Sequencing coverage metrics for targeted long-read sequencing assay.
(**A**) Average coverage depth for gene targets (n = 469) obtained for each participant in our undiagnosed spastic-ataxia cohort (n = 34 individuals). (**B**) Average coverage depth for participants (n = 34) obtained for each gene target on our targeted long-read sequencing panel; given space constraints, only the fifty genes with lowest and highest coverage are shown.
