# Supplementary material for: Targeted Long‐Read Sequencing as a Single Assay Improves the Diagnosis of Spastic‐Ataxia Disorders
Source: Ann Clin Transl Neurol. 2025 Feb 25;12(4):832–41. doi: 10.1002/acn3.70008 (PMC12040508; doi:10.1002/acn3.70008)
Supplement: Supplementary file 2 — Figure S2. Accuracy of targeted long‐read sequencing confirmed by analysis of positive control cases. [file ACN3-12-832-s004.pdf]

A Summary of known pathogenic variants in control cases

Participant	Diagnosis	Gene	Variant(s)	Original testing method	ONT LRS Result
35	FXTAS	*FMR1*	$(CCG)_{85}$	PCR and triplet-primed PCR	$(CGG)_{9}(AGG)_{1}(CGG)_{9}(AGG)_{1}(CGG)_{67}$
39	SCA3	*ATXN3*	$(CAG)_{73}$	PCR and fragment analysis	$(CAG)_{2}(CAA)_{1}(AAG)_{1}(CAG)_{1}(CAA)_{1}(CAG)_{71}$
36	SPG7	*SPG7*	c.1529C>T (p.Ala510Val) presumed homozygous	Targeted NGS panel	c.1529C>T in homozygosity
37	SCAR10	*ANO10*	c.132dup (p.Asp45Argfs*9); c.1219-1G>T	Targeted NGS panel	c.132dup (p.Asp45Argfs*9); c.1219-1G>T *in trans*
38	SCAR23	*TDP2*	c.425+1G>A; c.728del (p.Met243Argfs*3)	WGS	c.425+1G>A ; c.728del (p.Met243Argfs*3) *in trans*

B Identification of STR expansions in control cases

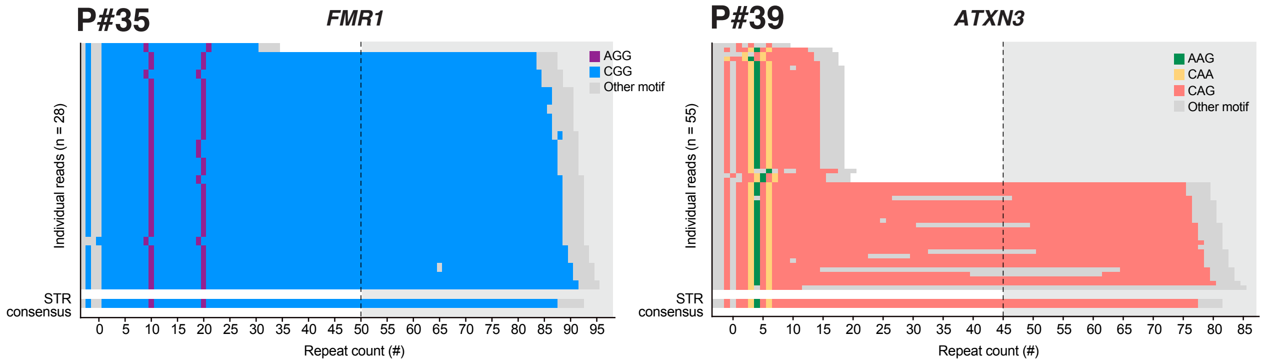

C Detection and phasing of sequence variants in control cases

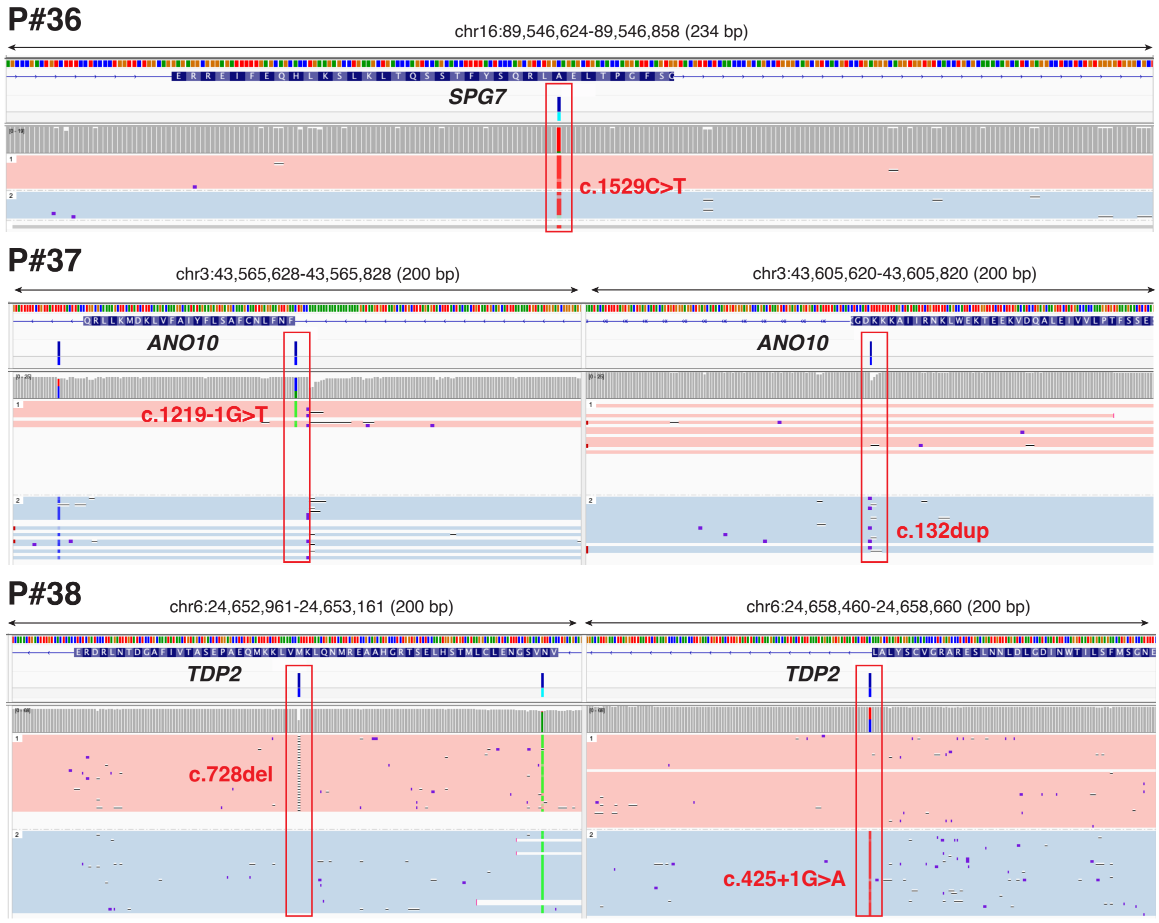

Supplementary Figure 2. Accuracy of targeted long-read sequencing confirmed by analysis of positive control cases. (**A**) Table provides an overview of analysis results for five spastic-ataxia patients with known genetic diagnoses who were included as positive controls. Glossary: FXTAS, fragile X tremor/ataxia syndrome; NGS, next generation sequencing; ONT LRS, Oxford Nanopore Technologies long-read sequencing; PCR, polymerase chain reaction; SCA, spinocerebellar ataxia; SCAR, spinocerebellar ataxia, recessive; SPG, spastic paraplegia; WGS, whole genome sequencing. (**B**) Sequence-bar plots show STR genotyping results for FMR1 (Patient #35; FXTAS) and ATXN3 (Patient #39; SCA3). Each individual long-read alignment is show separately and a consensus sequence for the expanded allele in each case is shown below (note: only one allele is present for Patient #35, as FMR1 is on chrX and the individual is male). (**C**) Genome browser view shows detection of known pathogenic sequence variants within SPG7 (Patient #36), ANO10 (Patient #37) and TDP2 (Patient #38). Alignments are phased into separate haplotypes (pink = haplotype 1; blue = haplotype 2), confirming the pathogenic variants for #37 and #38 are on alternative haplotypes (i.e. in trans).
