# Supplementary material for: Targeted Long‐Read Sequencing as a Single Assay Improves the Diagnosis of Spastic‐Ataxia Disorders
Source: Ann Clin Transl Neurol. 2025 Feb 25;12(4):832–41. doi: 10.1002/acn3.70008 (PMC12040508; doi:10.1002/acn3.70008)
Supplement: Supplementary file 3 — Figure S3. Detection and phasing of pathogenic sequence variants in four undiagnosed participants. [file ACN3-12-832-s006.pdf]

Detection and phasing of pathogenic sequence variants in four undiagnosed participants

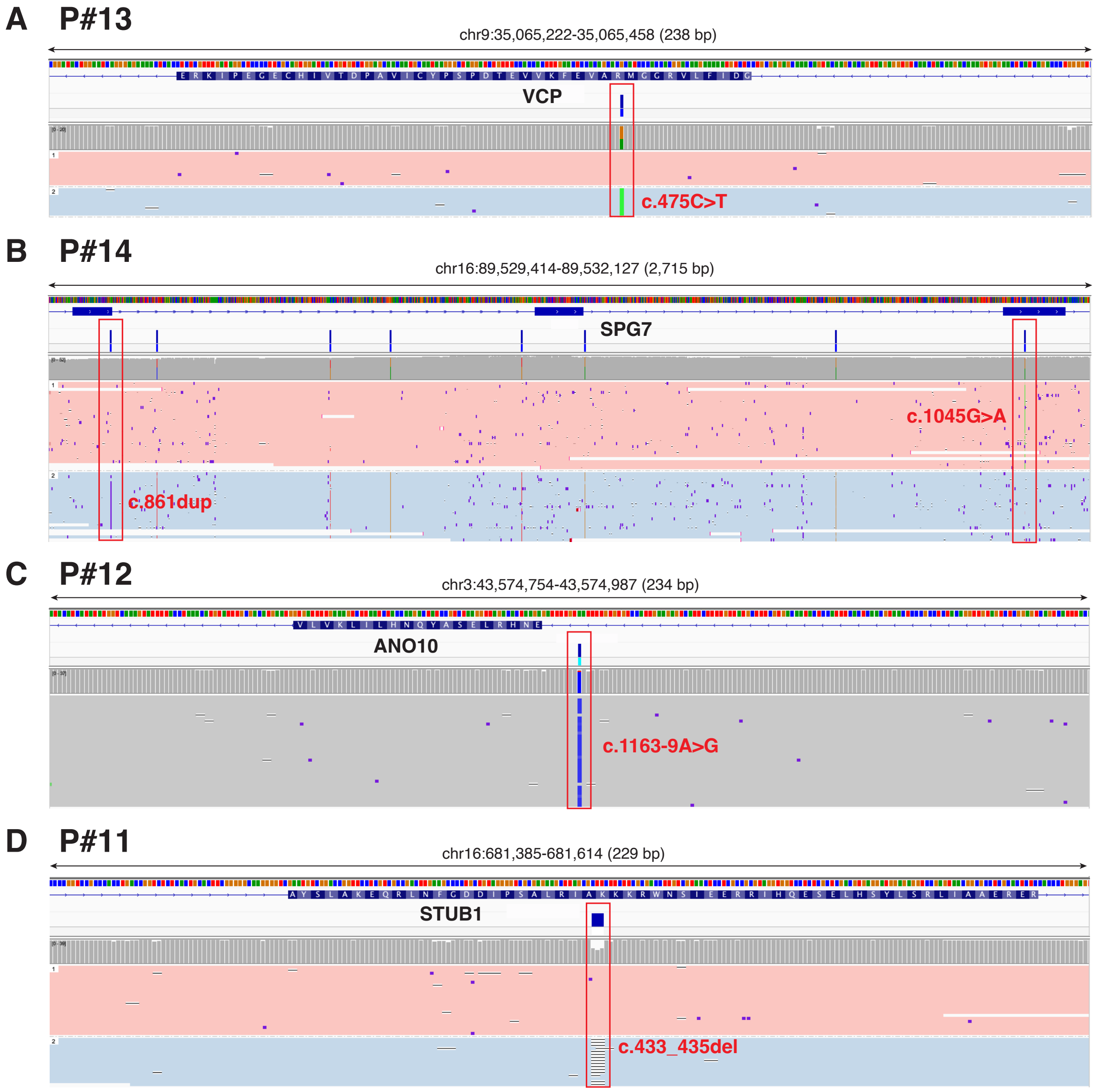

Supplementary Figure 3. Detection and phasing of pathogenic sequence variants in four undiagnosed participants. Genome browser view shows detection of pathogenic sequence variants within (**A**) VCP (Patient #13); (**B**) SPG7 (Patient #14); (**C**) ANO10 (Patient #12) and (**D**) STUB1 (Patient #11). Alignments are phased into separate haplotypes (pink = haplotype 1; blue = haplotype 2).
