# Supplementary material for: Targeted Long‐Read Sequencing as a Single Assay Improves the Diagnosis of Spastic‐Ataxia Disorders
Source: Ann Clin Transl Neurol. 2025 Feb 25;12(4):832–41. doi: 10.1002/acn3.70008 (PMC12040508; doi:10.1002/acn3.70008)
Supplement: Supplementary file 10 — Table S7. FGF14 STR expansion lengths on LRS and confirmatory flanking/repeat‐primed PCR. [file ACN3-12-832-s002.docx]

**Supplementary Table 7. *FGF14* STR expansion lengths on LRS and confirmatory flanking/repeat-primed PCR**

| **ID** | ***FGF14* GAA repeat length** | |
| --- | --- | --- |
|  | **ONT LRS** | **Confirmatory testing – F/RP-PCR** |
| 1 | 296/40 | 302/38 |
| 3 | 321/9 | 330/9 |
| 4 | 274/9 | 274/9 |
| 5 | 387/9 | 414/8 |
| 7 | 338/17 | 340/17 |

F/RP-PCR: flanking and repeat-primed PCR; ONT LRS: Oxford Nanopore Technologies long-read sequencing
