# Supplementary material for: Targeted Long‐Read Sequencing as a Single Assay Improves the Diagnosis of Spastic‐Ataxia Disorders
Source: Ann Clin Transl Neurol. 2025 Feb 25;12(4):832–41. doi: 10.1002/acn3.70008 (PMC12040508; doi:10.1002/acn3.70008)
Supplement: Supplementary file 13 — File S1. Genetic and clinical features for participants with nondiagnostic findings and variants of uncertain significance. [file ACN3-12-832-s011.docx]

**Supplementary File 1. Genetic and clinical features for participants with non-diagnostic findings and variants of uncertain significance**

**Biallelic *FGF14* GAA expansion (each <250 repeats)**

A patient of Scottish ancestry, presented with sporadic late-onset ataxia from age 68. The year prior to symptom onset, the patient had suffered a stroke with residual speech and swallow impairment. He had a single episode of vertigo at age 70, attributed to benign paroxysmal positional vertigo, but episodic features were not otherwise reported. Two years post symptom onset, he required use of a walker, and after a further year, commenced using a wheelchair. On examination, he had down-beat nystagmus, saccadic pursuit, dysmetric saccades, dysarthria, upper limb dysmetria, dysdiadochokinesia, heel-shin ataxia and an ataxic gait. Head impulse test was positive to the left. MRI demonstrated generalized cerebral and cerebellar atrophy, in addition to mineral deposition in the basal ganglia and a chronic infarct in the posterior limb of the left internal capsule. ONT LRS identified a biallelic *FGF14* STR expansion with 213/201 GAA repeats. There is a suggestion that biallelic GAA alleles <250 repeats might be associated with disease^1^ but will also require publication of further cases.

**Non-pathogenic *RFC1* STR expansions**

Two individuals had monoallelic *RFC1* (AAGGG)_n_ expansions of 483 and 440 repeats respectively (Figure 2A). Prior reports have identified that compound heterozygosity with a *RFC1* (AAGGG)_n_ expansion and a truncating variant can cause *RFC1*-CANVAS^2, 3^. However, ONT LRS sequencing data did not identify a second hit in either of these two cases.

**Sequencing variants of uncertain clinical relevance**

Variants in the *GCH1* and *PRRT2* genes were identified in unsolved participants (Supplementary Table 4). Despite follow-up examinations, the clinical relevance was unclear.

The *GCH1* variant carrier was a 66-year-old lady, of mixed Australian Indigenous and English/Irish/Scottish ancestry, who presented with progressive balance decline, dizziness and an upper limb tremor from age 63. There was no family history of similar features. On examination there were mild features of ataxia, with saccadic pursuit, horizontal gaze-evoked nystagmus, intention tremor, mild heel-shin ataxia and a wide-based gait. Head-impulse testing was negative. Sensory examination demonstrated mild distal sensory impairment in the lower limbs. There was a jerky tremor of the upper limbs, most notably with certain postures, suspected to reflect a dystonic tremor. There were no other extrapyramidal features. ONT LRS identified a variant in *GCH1* (c.671A>G, p.Lys224Arg), classified as likely pathogenic. Additional VUSs were identified in *KCNA1* (c.368G>A, p.Gly123Asp) and *TTBK2* (c.3485G>A, p.Arg1162His)*.*

The *PRRT2* variant carrier, a 59-year-old man, with British ancestry, presented with a complex neurologic disorder of ten-year duration. He had a family history of dementia in both his mother and maternal grandmother. Features included fasciculations, spasticity, hyperreflexia and mild distal weakness of upper and lower limbs, cerebellar features (gaze-evoked nystagmus, saccadic pursuit, dysmetric saccades, dysarthria and upper and lower limb ataxia), upper limb bradykinesia and a mild rest tremor. Gait was broad-based, with slow initiation and shuffling steps. He reported autonomic features (erectile dysfunction and urinary urgency) and although not formally tested, cognitive decline was suspected, with difficulties processing complex information, and a tendency to burst into laughter with cognitive loading, suggestive of pseudobulbar affect. MRI demonstrated diffuse cerebral involution without disproportionate cerebellar atrophy. NCS revealed a sensorimotor polyneuropathy, and EMG showed chronic neurogenic changes in limb and cranial nerve-innervated muscles. Motor evoked potentials revealed marked dysfunction of central motor pathways to all limbs. During investigation, the patient was also identified to have positive CASPR2 antibodies on blood testing, which were negative on CSF. This finding was of uncertain significance, but the patient was trialled on IVIg and mycophenolate without significant response. ONT LRS identified a variant in *PRRT2* (c.649dup, p.Arg217ProfsTer8), which was classified as pathogenic although is of uncertain clinical significance in this case. An additional VUS was identified in *SPTBN2* (c.6250G>A, p.Glu2084Lys).

**References**

1. Ouyang R, Wan L, Pellerin D, et al. The genetic landscape and phenotypic spectrum of GAA-FGF14 ataxia in China: a large cohort study. EBioMedicine. 2024 Apr;102:105077.

2. Benkirane M, Da Cunha D, Marelli C, et al. RFC1 nonsense and frameshift variants cause CANVAS: clues for an unsolved pathophysiology. Brain. 2022 Nov 21;145(11):3770-5.

3. Ronco R, Perini C, Curro R, et al. Truncating Variants in RFC1 in Cerebellar Ataxia, Neuropathy, and Vestibular Areflexia Syndrome. Neurology. 2023 Jan 31;100(5):e543-e54.
